# Randomised controlled trial of the new short-term online emotion focused training for self-compassion and self-protection in a nonclinical sample

**DOI:** 10.1007/s12144-018-9933-4

**Published:** 2018-07-30

**Authors:** Júlia Halamová, Martin Kanovský, Karolína Varšová, Nuriye Kupeli

**Affiliations:** 1 Institute of Applied Psychology, Faculty of Social and Economic Sciences, Comenius University in Bratislava, Mlynské luhy 4, 821 05 Bratislava, Slovakia; 2 Institute of Social Anthropology, Faculty of Social and Economic Sciences, Comenius University in Bratislava, Bratislava, Slovakia; 3 Marie Curie Palliative Care Research Department, Division of Psychiatry, University College London, London, UK

**Keywords:** Self-compassion, Self-criticism, Emotion-focused therapy, Randomized controlled trial, Experiment

## Abstract

The Emotion Focused Training for Self-Compassion and Self-Protection (EFT-SCP) is an intervention developed to increase skills of self-compassion and protective anger with the aim to decrease self-criticism. This novel intervention was developed on the basis of the latest findings on self-criticism from Emotion-focused therapy and previous programs cultivating compassion (namely Compassion Mind Training and Mindful Self-Compassion Program). According to existing research, simply cultivating self-compassion is not always sufficient in reducing self-criticism. Therefore, the EFT-SCP was designed to build self-compassion whilst developing protective anger to combat self-criticism. Our goal was to investigate the efficacy of this new, short-term, online EFT-SCP program in a non-clinical population. A randomized control trial was conducted with pre- and post-intervention measurements and two-month follow-up of self-compassion and self-criticism/reassurance. Convenience sampling was used to recruit participants through a snowballing technique on social media. A total of 123 participants were randomly allocated to the EFT-SCP intervention or to a control condition. The intervention group were instructed through emails to complete an EFT-SCP task every day for 14 consecutive days. The control group did not complete any tasks. Out of 123 participants, 31 from intervention group and 20 from control group completed all measurements. There was a significant effect of the EFT-SCP on increasing self-compassion and self-reassurance scores as reported at two-month follow-up. The EFT-SCP was also effective at reducing self-uncompassionate responding and self-criticism (specifically Hated self) with changes evident at two months post-intervention. These findings are encouraging and suggest that interventions designed to enhance self-compassion and decrease self-criticism can be delivered to broader populations without the direct contact with mental health professionals.

## Introduction

Self-critical and self-compassionate inner dialogues are the very opposite ways of relating to the self. Self-criticism was described by Blatt and Zuroff (1992) as constant and harsh self-scrutiny and evaluation and feelings of unworthiness, shame, inferiority, failure, and guilt. On contrary, self-compassion as well as compassion towards others were characterised by Strauss et al. (2016) as being aware of own suffering and the universality of human suffering, feeling emotional resonance with the suffering self, tolerating unpleasant feelings connected to the suffering, and being motivated to relieve own suffering.

According to recent research, high levels of self-criticism and low levels of self-compassion are associated with psychopathology (Blatt 2004; Falconer et al. 2015; Shahar et al. 2012) while high levels of self-compassion and low levels of self-criticism are important factors of a happy life and well-being (MacBeth and Gumley 2012; Zessin et al. 2015). Specifically, research has shown a relationship between high self-criticism and social anxiety, depression, post-traumatic stress disorder, behaviors that involve inflicting self-harm, suicidal tendencies (O’Connor and Noyce 2008), bipolar disorder, schizophrenia, eating disorders and borderline personality disorder (Meares et al. 2011). Therefore self-compassion and self-criticism are clinically significant and research on interventions influencing their levels is of great importance for clinical practice.

Several interventions have been developed to help people to cultivate compassion and alleviate self-criticism. According to Kirby (2016), out of the eight established compassion-based intervention programs, Compassionate Mind Training (Gilbert 2009, 2010) is the most evaluated intervention. Another programme which is becoming increasingly popular is The Mindful Self-Compassion training (Kirby 2016), which has over 200 trained teachers listed on its programme directory.

### Interventions Developed to Cultivate Self-Compassion

#### Compassion Mind Training

Compassionate Mind Training (CMT) was developed on the basis of compassion focused therapy (CFT; Gilbert 2010), which aims to balance the three affect regulation systems: the protection (against threat), resource-seeking (in order to achieve and strive), and soothing systems (for connectedness). CMT was designed to teach participants skills of compassion by improving accessibility to the affiliation-focused affect system and thus increasing the ability to self-soothe at times of stress. Commonly, CMT is delivered in one to two hour sessions over 6 to 12 weeks and group meetings take place once or twice a week. According to previous research, some studies suggest that CMT has a positive impact either on self-criticism (Gilbert and Procter 2006) or self-compassion (Gilbert and Irons 2004), whilst others report an effect of CMT on both self-compassion and self-criticism (Judge et al. 2012; Matos et al. 2017).

#### The Mindful Self-Compassion Program

The Mindful Self-Compassion program (MSC) was developed by Neff and Germer (2013) as a method to cultivate the skills of self-compassion. MSC builds upon Neff’s (2003) definition of self-compassion and the six bipolar primary components of self-compassion: Self-Kindness versus Self-Judgment, Common Humanity versus Isolation, and Mindfulness versus Over-identification. Self-kindness is related to being kind to oneself while self-judgement relates to being harsh or even cruel towards the self. Common humanity is linked to experience of suffering shared by all human beings whereas Isolation is associated to being alone in suffering and cut off from other people. Mindfulness is associated to being aware of painful experiences without judgement whereas Over-identification refers to being absorbed by one’s own pain. The program involves performing exercises in groups. In this program, participants meet as a group for 2–2.5 h with a certified MSC trainer once a week for eight weeks and are instructed to practice daily exercises at home. So far, there is some support demonstrating that MSC is effective in enhancing self-compassion in clinical and nonclinical samples (e.g. Friis et al. 2016; Neff and Germer 2013).

### Emotion-Focused Therapy

The interventions presented so far are well-established compassion-based interventions. However, Emotion-focused therapy, one of empirically supported treatments by The American Psychological Association (Greenberg 2011), has been shown to be effective in decreasing self-criticism (e.g. Elliott et al. 2004) and increasing self-compassion (e.g. Timulak 2015). Therefore, Emotion-focused therapy theory and its techniques may be a useful source for reducing self-criticism in nonclinical populations as a preventative intervention to reduce the risk of psychopathology.

Self-criticism is one of the markers of Emotion-focused therapy which is addressed by using the two-chair dialogue between part of self as critic and part of self as experiencer. The purpose of two-chair dialogue is to enact self-critical attacks in order to evoke the painful feelings, highlight the negative self-treatment, access underlying core painful emotions which the negative self-treatment evokes and finally transform these core painful emotions by activating protective anger and self-compassion (Elliott et al. 2004; Greenberg 2011; Shahar et al. 2017; Timulak 2015).

In Emotion-focused therapy, therapists coach clients towards primary adaptive emotions that “are attended to and expressed in therapy in order to access the adaptive information and action tendency to guide problem solving” (Elliott et al. 2004, pg. 31). As previously mentioned, in dealing with self-criticism, there are two primary adaptive feelings which therapist guide clients to address. First of them is protective or empowering anger which people feel while they are mistreated and it motivates them to stand against dismissive other (in this case self-critic), take assertive actions to set boundaries, protect oneself and stop the mistreatment. Another desirable primary adaptive emotional response in working with self-criticism is self-compassion. Generally, people feel compassion when they face suffering and it motivates them to take actions to alleviate the suffering. In Emotion-focused therapy, clients experience genuine compassion towards the self which is suffering by one’s own self-critical processes (Timulak 2015).

To date, studies indicate that self-criticism can be transformed by accessing both protective anger at mistreatment and self-compassion during suffering (Pascual-Leone and Greenberg 2007; Timulak 2015**;** Timulak and Pascual-Leone 2014). There are examples of research studies, outside of studies which have used the EFT as a psychotherapeutic treatment, which have demonstrated that EFT principles are effective in reducing self-criticism and improving self-compassion. For example it relates to the one-session study of Shahar et al. (2012) which examined the efficacy of the two-chair dialogue, on self-criticism, self-compassion and self-reassurance on highly self-critical participants. As expected, results showed significant changes in self-compassion, self-reassurance, and self-criticism.

So far, the findings about effectiveness of this treatment of self-criticism were supported by research studies conveyed during psychotherapy. What is more, similar findings about the importance of protective anger and self-compassion in combating self-criticism were reported by Whelton and Greenberg (2005), and Kelly et al. (2009) in research settings beyond psychotherapy sessions. Those participants who presented more contempt, shame, disgust and less resilience and anger in response to their self-criticism were more self-critical (Whelton and Greenberg 2005). All participants demonstrated self-criticism but only highly self-critical participants were not able to stand against their own self-criticism and collapsed under self-attacks from their own critic or they met them with submission. Similarly, participants in a study using two different self-help interventions benefited more from an attack-resisting condition and self-compassionate condition compared to a control condition (Kelly et al. 2009). Kelly et al. (2009) did not combine self-soothing and attack-resisting interventions together and therefore they were not able to test the effects of such a combined treatment. Authors concluded that future research should test an intervention combining self-compassionate and self-attack resistance learning.

Taken together, these findings suggest that increasing the ability to be self-compassionate as well as to be self-protective might be essential to reducing self-criticism. According to Gilbert and Irons (2004) highly self-critical people have difficulties with self-compassion and may benefit from practicing self-compassion because it can lower their level of self-criticism. However, findings from Emotion-focused therapy showed that learning self-compassion might not be enough for combating self-criticism but that it should be combined with learning protective anger. Currently, compassion-based interventions (Kirby 2016) have focused primarily on developing compassion and self-compassion, but these programs do not directly address the need to build assertive reactions that would elicit protective anger to stand up against own self-critic. Therefore, we have used recent developments in the field of interventions that support self-compassion (Compassion Mind Training, and Mindful Self-compassion program) and Emotion-focused therapy to develop a new self-compassion intervention. There have also been attempts to adapt components of EFT into a set of exercises or tasks to be completed outside of the therapeutic sessions (Berg 2012; Greenberg and Warwar 2006; Halamová 2013). The current study will exploit these adaptations to pilot a novel online-based intervention based on emotion-focused therapy.

### Aims

The primary aim of the present study was to evaluate the immediate and longer term impact of a 14-day internet-based version of the Emotion Focused Training for Self-Compassion and Self-protection (EFT-SCP) on self-compassion, self-criticism and their dimensions in a non-clinical population.

## Methods

### Measures

Self-criticism/reassurance was assessed using the **Forms of Self-Criticism/Reassuring Scale** (FSCRS; Gilbert et al. 2004). The FSCRS is a 22-item measure requiring participants to rate a selection of positive and negative statements on a 5-point Likert scale (“Not at all like me” to “Extremely like me”). Items include “I am easily disappointed with myself” and “I am gentle and supportive with myself”. Positive items reflect the ability to self-reassure (referred to as reassured-self) and negative items indicate self-critical thoughts and feelings (split into subscales of inadequate-self and hated-self). This scale, its psychometric properties and its factor structure have been validated in various clinical as well as nonclinical samples in different countries (e.g. Baião et al. 2015; Halamová et al. 2017a; Kupeli et al. 2013).

Self-compassion was assessed using the **Self-Compassion Scale** (SCS; Neff 2003). The SCS measures six components of self-compassion experienced during perceived difficulty. The scale consists of 26 items rated on a 5-point Likert-type scale (1 = almost never; 5 = almost always). The scale consists of six subscales that measure the degree to which individuals display self-kindness against self-judgment, common humanity versus isolation, and mindfulness versus over-identification. The psychometric properties of the scale have been validated in different countries (e.g. Halamová et al. 2017b). Some recent studies on the SCS demonstrated that negative and positive subscales of SCS should be calculated separately and should not be summed as a single score (Brenner et al. 2017; López et al. 2015). Therefore, for the purpose of this study, the combined score of the positive constructs (self-compassionate responding: self-kindness, humanity and mindfulness) and the combined score of the negative constructs (self-uncompassionate responding: self-judgement, isolation and over-identification) was used.

### Recruitment

Participants were recruited from the general community through social media, social networking sites and health and well-being forums. As a gesture of gratitude, those who completed all phases of the study were entered into a prize draw to win a tablet. The data collected was in accordance with the ethical standards of the institutional research committee and with the 1964 Helsinki declaration and its later amendments or comparable ethical standards.

### Participants

**Fig. 1:**
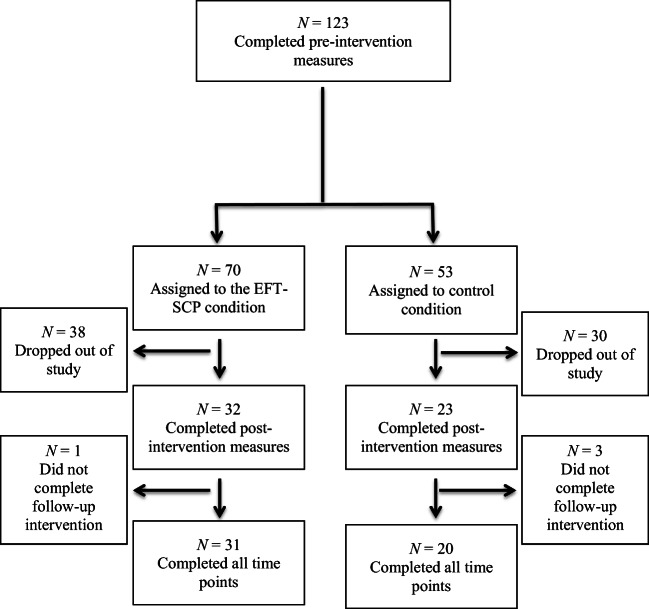
Flow chart for the number of participants who completed each phase of the study and attrition

A total of 123 participants completed the pre-intervention measures and from this sample, 70 were randomly allocated to the intervention group and 53 were assigned to the control condition (see Fig. 1 for study attrition information). Once the participants completed the pre-intervention measures, the first eleven participants were allocated to the intervention condition and the next set of eight participants was allocated to the control condition. This was done until all participants were allocated into the two conditions. We expected that the intervention group would have more attrition rate hence why more participants were assigned to this group. From this sample, 32 participants from the intervention group and 23 participants from the control group completed the post-intervention measures. Out of the participants assigned to the EFT-SCP group, 31 completed the two-month follow-up and 20 of the 23 participants of the control group completed the follow-up measures. The final control group consisted of 17 women and 3 men with a mean age of 25.35 years (*SD* = 6.32) and the intervention group consisted of 26 women and 5 men with mean age of 33.73 years (*SD* = 16.00). All participants were Caucasians and Slovaks. In terms of education, 33.3% were graduates from university, 24% were undergraduates, 39.6% had secondary education and 3.1% had basic education.

### Procedure

All participants completed demographic information and baseline measures and were then randomly allocated to the intervention and control groups. The control group were not provided with any additional instructions until 14 days after completing the baseline measures when they received an email to a link to the online self-report measures and this process was repeated for the two-month follow-up. Participants were given three days to complete the measures.

Participants assigned to the EFT-SCP condition were instructed to complete a daily EFT-SCP task for 14 consecutive days. Each participant assigned to the intervention group received an email prompting them to complete the EFT-SCP task and each participant received the same task each day (i.e., tasks were not randomised). Each email was presented in the same format which consisted of a short introduction in the form of psychoeducation which explained the intended impact of the task in order to motivate participants to do it, the instruction about how to complete the task, a description of the task, a link to an online document for participants to complete the task, and questions about the task designed to encourage participants to reflect on their experience (adherence check). Following our previous experience of conducting interventions, we applied a simple formula: explain what and why people should do it, let them perform the task and ask them to reflect on the task they completed to increase and fix the impact of the exercise. In order to even deepen the emotion processing and experience of the intervention, we have used expressive writing (Pennebaker 2017; Pennebaker and Beall 1986) as a tool for delivering all exercises. The additional function of the post-task questions was a manipulation check to ensure participants completed the appropriate task and it included the same four free-text response items after each exercise:

1. How did you find completing the exercise? (General feedback about the exercise)
2. How did you feel about it? (Emotion related feedback)
3. What did you realize during this exercise? (Cognition related feedback)
4. What do you take from this exercise into your everyday life? (Behaviour related feedback)

If the participant had not completed the exercise, they were sent an email reminder.

The tasks were selected from different exercises related to self-compassion and self-criticism available from previous publications (e.g., Berg 2012; Gilbert 2009; Gilbert 2010; Germer 2016; Germer and Neff 2013; Greenberg and Warwar 2006; Halamová 2013; Neff 2017; Rockman and Hurley 2015). Each exercise was selected by consensus of our research team using the criteria of capturing the core elements of the EFT process of change, the expected impact on participants and their motivation to complete them. The exercises were translated and tailored by experts (JH and KV). All of the exercises were presented in the form of expressive writing. Participants were instructed to spend at least 15 min per day by doing exercises and at least 15 min by writing about their experience with the particular exercise. The intervention was accessible on any computer or smartphone via a link on the day the email was sent. The exercises were selected and presented to participants in the following order:

- *Day 1*: How would you treat a friend? (Gilbert 2010, p. 48; Neff 2017; Rockman and Hurley 2015, p. 5). This task was designed to evoke insight into different approaches people commonly use for treating friends and themselves during adversity and then help them to turn the more compassionate language, people usually have for their friends, towards themselves.
- *Day 2*: Compassionate letter to myself (Gilbert 2010, p. 81; Neff 2017; Rockman and Hurley 2015, p. 22). This task involved writing about what you don’t like about yourself and how this makes you feel. The second part of the task was to imagine a compassionate friend and using their perspective, write about how this friend views your flaws.
- *Day 3*: Letting go of a painful memory from your childhood (1st part). This task involved writing a letter from yourself as a child expressing your past pain (Halamová 2013, p. 69).
- *Day 4*: Letting go of a painful memory from childhood (2nd part). This task involved writing a letter from one’s own perspective as an adult to themselves as a child and it was designed to enable one to express compassion and protective anger towards themselves as a child (Halamová 2013, p. 69)
- *Day 5*: Letting go of a painful memory from childhood (3rd part). This task was to read the letter from the adult as they would do if they were a child again and to respond from the child’s perspective expressing their emotions and needs. The final exercise of this task was to respond to the child’s needs from the adult perspective (Halamová 2013, p. 69)
- *Day 6*: Expressing protective anger (modified from Berg 2012, p. 19; Greenberg and Warwar 2006, p. 193–4; Halamová 2013, p. 57). This task involved recalling an event when someone was critical towards you or was shaming you and to imagine how your close friend would defend or protect you, then reformulate the same protective response from your perspective to the self. This task was designed to enable participants to express their protective anger.
- *Day 7*: Expressing compassion towards the self (modified from Berg 2012, p. 21; Greenberg and Warwar 2006, p. 194; Halamová 2013, p. 59). This task involved recalling a self-critical event and imagining that this had happened to a vulnerable child. Participants were instructed to be compassionate towards the child and then turn the same compassionate response towards the self.
- *Day 8*: Self-compassionate mirror. This task required participants to look in the mirror at the end of the day and be self-compassionate about pleasant or unpleasant events which may have occurred during the day followed by an expressive writing task to write about this experience. This task was designed to promote the experience of self-compassion (inspired by Petrocchi et al. 2017).
- *Day 9*: Compassionate friend (Gilbert 2009; Rockman and Hurley 2015, p. 35). This task involved imagining that a compassionate friend is coming to visit you and when they arrive, they tell you all the things you need to hear at this moment in your life and they present you with a gift that has a special meaning for you.
- *Day 10*: Self-compassion break (Neff 2017; Rockman and Hurley 2015, p. 7). This task involved recalling a stressful experience and putting your hand on your heart and saying to yourself that it is a moment of suffering, reason that other people suffer too and that you can still be kind to yourself. Participants are then instructed to write about their experience.
- *Day 11*: Self-compassionate language (Rockman and Hurley 2015, p. 8). During this task participants were instructed to list their typical criticisms and reframe them into compassionate words towards themselves.
- *Day 12*: Self-compassion in daily life (Germer 2016). This task involved searching for new ways to be more self-compassionate on a physical, emotional, rational, social, and spiritual level and writing about these new approaches.
- *Day 13*: Self-compassion in everyday life. During day 14, participants were instructed to practice the new self-compassionate ways they identified on day 13 and write about their experience of using these new approaches in the evening.
- *Day 14*: Thanksgiving. The final task involved making a list of as many things as possible that you are grateful for in your life (this task is similar to the Appreciation exercise by Gilbert 2010 and Appreciating Yourself by Germer and Neff 2013 and Rockman and Hurley 2015)

Following the final exercise, participants were instructed to complete the post-intervention measures and this was repeated at the two-month follow-up.

### Data Analyses

For data analyses, we used SPSS Statistics-20, and for the statistical processing, program R (Version 3. 4. 0, R Core Team 2013), and the package nparLD (Noguchi et al. 2012) were used.

Factorial designs of this type (split-plot design) are usually analysed by means of parametric procedures (ANOVA). However, the assumptions of parametric methods such as homoscedasticity, normality, or absence of outliers are seldom met in practice. Many classical non-parametric alternatives (Wilcoxon-Mann-Whitney test, Kruskal-Wallis test, Wald-type statistics) perform poorly for small sample sizes, heteroscedascity and unbalanced designs (when the size of control and experimental sample is different; see Brunner et al. 1999; Brunner et al. 2002; Brunner et al. 2016). Mathematically, our dependent variables are raw scores of ordinal items, normal distribution cannot be assumed (in fact, many of them display non-normal distribution in Shapiro-Wilk tests). Moreover, our intervention design practically excludes equal variances of control and experimental groups (see Tables 2 and 4). Therefore our data are heteroscedastic, as it should be: intervention usually decreases variance in its group. There are well-justified statistical reasons to use nonparametric heteroscedastic methods for our statistical analyses. We will report ANOVA-type statistics (Brunner et al. 2016) from non-parametric rank-based test for longitudinal data, and relative effects which can serve as effect size measures. The relative effect can be regarded as the probability that a randomly chosen observation from the treatment group takes on larger values than an observation randomly chosen from the mean distribution function. Therefore a relative effect significantly higher (for increasing effect) or lower (for decreasing effect) than 0.50 indicates that an intervention was effective. ANOVA-type statistics (ATS) performs well even for small sample sizes and unbalanced designs (Brunner et al. 2002).

In most cases when a split-plot design with repeated measures is conducted, it is mainly of interest to investigate an interaction between groups (factor G) and time (factor T). In our split-plot design, there is a control group without intervention (group 1) and the active intervention group (group 2), therefore the distribution functions at the start of the trial (time point 1) are identical because the subjects were randomly assigned to the two groups of factor G. Then, an effect of the active intervention will produce non parallel time curves of the measurements. This means that there should be a significant interaction between factor G and factor T if the intervention is effective. We hypothesize that our intervention will be significantly effective if and only if the interaction between group (control vs. intervention) and time (three time points) will be significant: therefore just the significant difference between control and experimental group or between time points will not do. Main factorial effects (difference between groups regardless of time, or difference among time points regardless of groups) are of no use here, so we will not report them.

## Results

Before the analysis, to ensure random assignment of participants to the two groups was successful, we checked for possible differences between control and intervention groups on pre-intervention scores. Since distributions and variances of groups are almost equal, we used the nonparametric Wilcoxon-Mann-Whitney test. Results showed that the groups did not differ on baseline scores for the SCS (*p*-values ranged from 0.22 to 0.83) and the FSCRS variables (*p*-values ranged between 0.15–0.71). In addition, results demonstrated that no significant differences were present between completers and drop-outs, for all SCS subscales and for all FSCRS subscales (*p*-values ˃ 0.248).

**Fig. 2:**
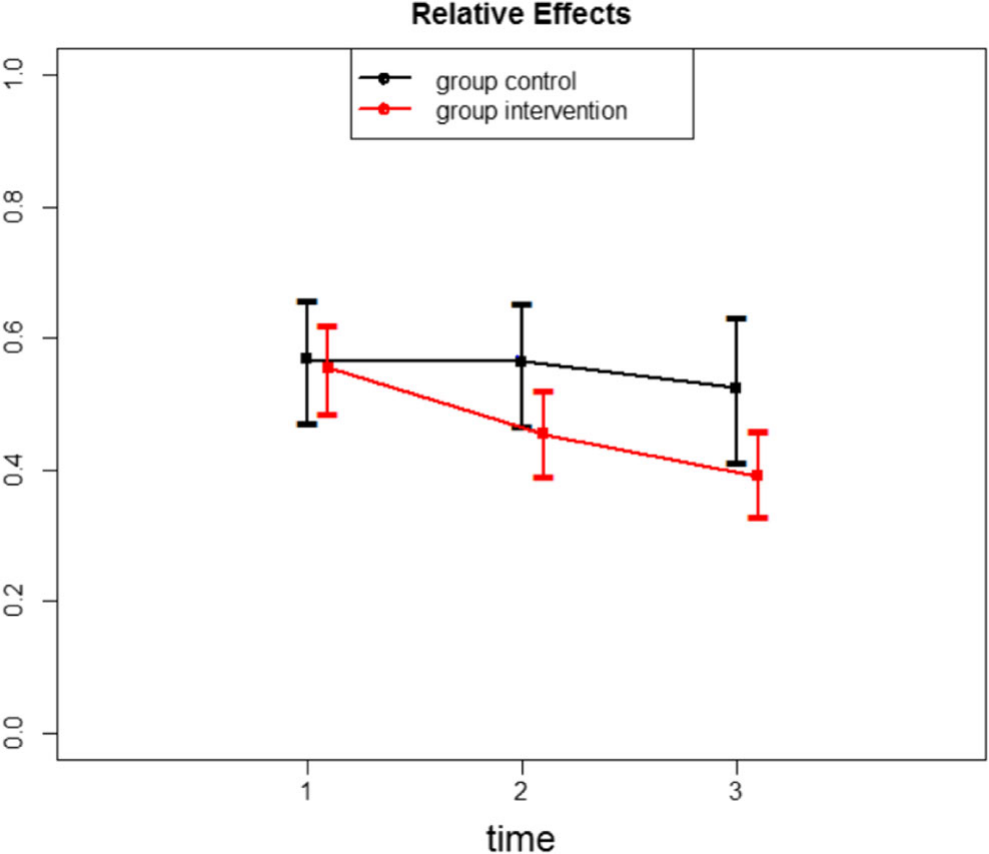
Relative effects for the Hated Self of the FSCRS scale

**Fig. 3:**
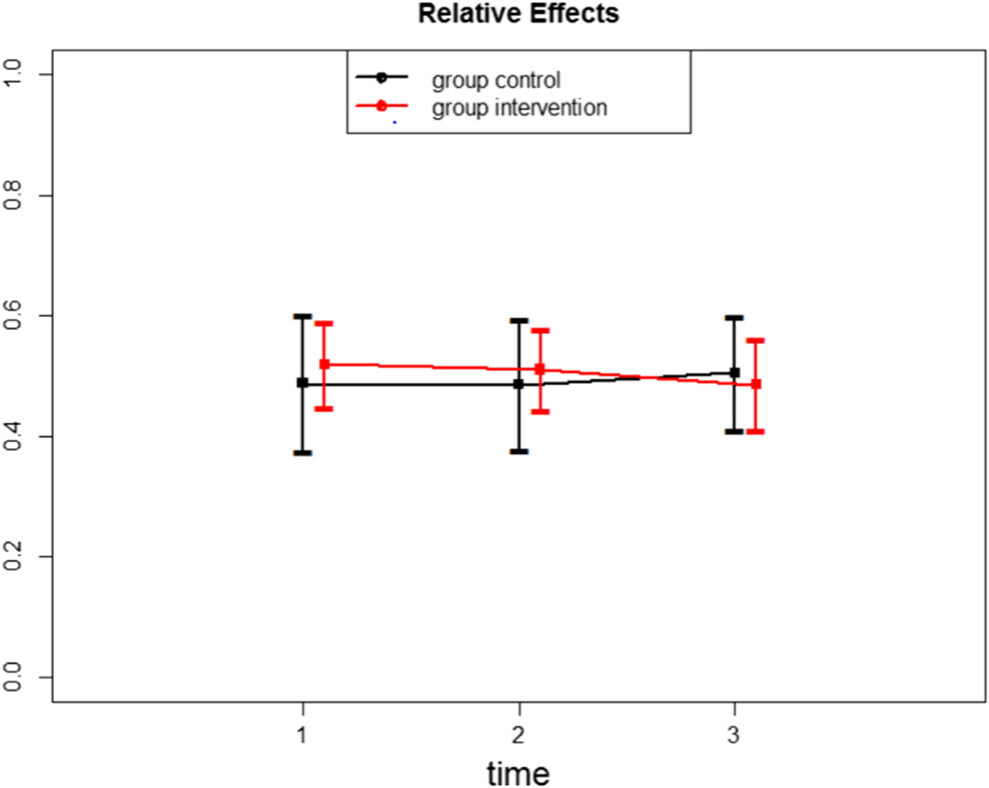
Relative effects for the Inadequate Self of the FSCRS scale

There was no effect of the intervention on immediately post-intervention scores of self-compassion and self-criticism/reassurance. However, the intervention had a significant effect on the FSCRS subscales, Reassured Self and Hated Self but no effect of the intervention on Inadequate Self (Table 1) as reported at the two-month follow-up. Relative effects with their confidence intervals for each group and time point (Table 2) allow a more detailed insight: for example, if we compare relative effects of Hated Self (Fig. 2) with Inadequate Self (Fig. 3), we can clearly see the significant and persistent change on Fig. 2 and no effect on Fig. 3.

**Table 1:** Results for interaction effects of the FSCRS scale

	ATS		
	F	df	p
FSCRS Reassured-Self	4.51	1.82, ∞	0.013*
FSCRS Inadequate-Self	0.61	1.60, ∞	0.509
FSCRS Hated-Self	4.97	1.54, ∞	0.013*

*Significant at 0.05 level

**Significant at 0.01 level

***Significant at 0.001 level

**Table 2:** Relative effects, their confidence intervals and variances of the FSCRS scale

		Relative effect	Confidence Interval	Variance
FSCRS Reassured-Self				
Control	Pretest	0.53	0.43–0.63	0.139
	Posttest	0.52	0.41–0.62	0.150
	Follow-up	0.53	0.42–0.63	0.151
Intervention	Pretest	0.58	0.49–0.64	0.072
	Posttest	0.48	0.41–0.56	0.073
	Follow-up	0.39*	**0.33 – 0.47**	0.068
FSCRS Inadequate-Self				
Control	Pretest	0.49	0.37–0.60	0.181
	Posttest	0.49	0.38–0.59	0.162
	Follow-up	0.51	0.41–0.60	0.122
Intervention	Pretest	0.52	0.45–0.59	0.070
	Posttest	0.51	0.44–0.58	0.062
	Follow-up	0.49	0.41–0.56	0.079
FSCRS Hated-Self				
Control	Pretest	0.57	0.47–0.66	0.118
	Posttest	0.56	0.47–0.65	0.119
	Follow-up	0.52	0.41–0.63	0.166
Intervention	Pretest	0.56	0.49–0.62	0.063
	Posttest	0.45	0.39–0.52	0.056
	Follow-up	0.39*	**0.33 – 0.46**	0.057

*Significant at 0.05 level

There was an effect of the intervention on the two subscales of the SCS scale separately (positive and negative items) as well (Table 3) as reported at two-month follow-up. Relative effects with their confidence intervals for each group and time point are presented in Table 4.

**Table 3:** Results for interaction effects of the SCS scale

	ATS		
	F	df	p
SCS positive – Self-compassionate responding	3.52	1.81, ∞	0.034*
SCS negative– Self-uncompassionate responding	9.61	1.49, ∞	0.001***

*Significant at 0.05 level

**Significant at 0.01 level

***Significant at 0.001 level

**Table 4:** Relative effects, their confidence intervals and variances of the SCS scale

Group	Time point	Relative effect	Confidence Interval	Variance
SCS positive – Self-compassionate responding				
Control	Pretest	0.47	0.37–0.58	0.144
	Posttest	0.49	0.39–0.59	0.139
	Follow-up	0.49	0.38–0.60	0.157
Intervention	Pretest	0.41	0.38–0.51	0.059
	Posttest	0.52	0.45–0.58	0.058
	Follow-up	0.60*	**0.52 – 0.68**	0.091
SCS positive – Self-uncompassionate responding				
Control	Pretest	0.48	0.38–0.58	0.149
	Posttest	0.47	0.37–0.57	0.144
	Follow-up	0.45	0.36–0.55	0.121
Intervention	Pretest	0.46	0.39–0.54	0.076
	Posttest	0.51	0.44–0.58	0.060
	Follow-up	0.59*	**0.52 – 0.66**	0.061

*Significant at 0.05 level

## Discussion

The present study examined the immediate and enduring effects of a newly developed 14-day internet-based training of The Emotion Focused Training for Self-Compassion and Self-Protection (EFT-SCP) on self-compassion and self-criticism.

Performing exercises to cultivate the skills of self-compassion and self-protection for 14 days were found to improve self-compassion and self-criticism/reassurance as measured by the SCS (Neff 2003) and FSCRS (Gilbert et al. 2004), respectively. The findings suggest that the EFT-SCP has a lasting effect on self-compassionate responding, Reassuring self and Hated self and self-uncompassionate responding. These findings are promising because previous research on the two-chair EFT approach from Shahar et al. (2012) showed significant changes only on Inadequate self but not Hated self. Nevertheless, Hated self is considered to be more impervious to change in brief interventions because it is related to hatred and disgust over self accompanied by desire to cause pain to self (Gilbert et al. 2004). Our findings also suggest that these changes were more stable in time compared to the study of Shahar et al. (2012) where a reduction in Hated self occurred during the session but then the score increased again.

According to previous research, the online 14-day version of MSC (Neff and Germer 2013) resulted in long-term increases in self-compassion and self-reassurance (Halamová et al. 2018a), while an online 13-day version of CMT (Gilbert 2010) decreased self-criticism and self-uncompassionate responding with effects lasting at least two months (Halamová et al. 2018b). As previously advocated by Kelly et al. (2009), participants might benefit from a combination of the two approaches. Our present study combines these two approaches with the latest findings of an emotion-focused therapy perspective and has demonstrated that EFT-SCP intervention can address self-protection and self-compassion simultaneously and with possibly greater and longer impact on self-criticism. It confirms and supports our hypotheses that an intervention designed to target affect regulatory processes will aid individuals to cultivate self-compassion, enhance self-protection and decrease self-criticism.

Similar to previous research (Kelly et al. 2009; Shahar et al. 2012; Whelton and Greenberg 2005), our study showed that there is inner dialogue within the self related to self-criticism and different parts of self need to be explored more specifically (thoughts, emotions, needs, and motivations) in order to develop more self-compassion from critical part and more self-protection from experiencer part of self. Therefore, it is possible that the EFT-SCP was effective because it enabled one to build self-compassion whilst developing protective anger to combat self-criticism. As previously suggested, it may not be sufficient to learn to be compassionate towards one’s own self-critic, but one must also be assertive and express protective anger against self-criticism (Whelton and Greenberg 2005).

The findings of the present study are promising as self-compassionate interventions enhancing self-compassion and alleviating self-criticism can be developed using a similar, online, easy-to-administer format to target many people worldwide without any contact with a health professional. Contrary to previous findings (Elliott et al. 2004; Greenberg 2011; Kelly et al. 2009; Shahar et al. 2012; Whelton and Greenberg 2005), our study was provided without any direct contact of participants and mental health workers which is even more encouraging.

### Limitations and Future Directions

The main limitation of the study is that the present findings apply only to non-clinical populations and hence we do not know whether patients with psychopathology would be willing to do the tasks or experience any benefits from the intervention. In future, we recommend testing the effects of this intervention with clinical populations as there are already very promising results with even physiological gains as an impact of the group version of EFT-SCP intervention in a student population (Halamová et al. 2018c).

Another limitation is a non-treatment control condition. Consequently, all effects could be possibly attributed to the demand effects because receiving some kind of treatment might encourage participants in the intervention group to indicate that there was some kind of improvement simply because they believe that this is was the aim of the study. Additionally, participants were aware of the expected outcomes of the intervention so they could consciously or unconsciously modify their reports in order to meet the expectations.

Although, participant attrition is a common problem in web-based intervention studies (e.g. Richards and Richardson 2012) our study is comparable to Mitchell et al. (2009), who reported an attrition rate of 83% at three months. In our study the attrition rate was 66% and this suggests that more than half of the sample were engaged with the study. On the another hand, the self-selection of highly motivated people who completed all EFT-SCP exercises, makes the findings applicable to individuals interested in the research or the specific topic and can lead to bias of self-selection in measurement.

As this study recruited a sample from the general population and from one country, these findings cannot be generalised to a clinical population and to other countries without further evaluation with people with psychological morbidity worldwide which should be targeted in the following research studies in future.

As the research relied on self-report measures of self-compassion and self-criticism, it could potentially be biased for social desirability responding. Therefore, future research should assess outcomes using objective assessments such as physiological measures.

Notwithstanding these limitations, our study offers further understanding of self-critical and self-compassionate processes outside a psychotherapeutic setting as well as outside of any direct contact with any mental health professional.

### Conclusion

A short and web-based version of the newly developed The Emotion Focused Training for Self-Compassion and Self-Protection (EFT-SCP) has significantly increased self-compassion and self-reassurance and decreased self-uncompassionate responding and self-criticism (Hated self) with effects evident two months pot-intervention. Results suggest that interventions like this may also be used as a preventative tool to reduce the risk of developing psychopathology. These findings are encouraging and suggest that interventions designed to enhance self-compassion and decrease self-criticism can be delivered to broader populations without the direct contact with mental health professionals. This is particularly relevant to those who may be unable to access or be ashamed to contact a mental health care provider.
